# The Pathology of Trichinosis1Read before the Central New York Medical Association, Buffalo, N. Y., October 16, 1894.

**Published:** 1895-01

**Authors:** Frank J. Thornbury

**Affiliations:** Buffalo, N. Y.


### THE PATHOLOGY OF TRICHINOSIS.[1]

By FRANK J. THORNBURY, M. D., of Buffalo, N. Y.

MY OBSERVATIONS include a study of 1,000 cases of trichinosis observed in swine in my work as supervising microscopist in the Bureau of Animal Industry. During the eleven months in which these observations were made, 197,000 hogs have been inspected and from them 400,000 slides prepared, or about 1,300 slides daily. Time will permit only a synopsis of the lengthy article which I have written.

Trichinosis often occurs epidemically in herds of swine. This is a fact which I have repeatedly substantiated. Age is a predisposing cause of infection; old hogs are more liable to be diseased. The parts of the carcass most frequently invaded by this parasite I have found to be the diaphragm, neck and loin respectively. I have statistical observations covering several hundred cases which demonstrate this fact. The encapsulation of trichinæ is a subject which I have investigated somewhat in detail. Often the parasites are enveloped separately, although I have seen as many as eight trichinæ in a single capsule. When a specimen is recently invaded the parasites are found unencapsulated and sometimes *in motion*. This motion may be enhanced by applying heat to the slide, when

1. Read before the Central New York Medical Association, Buffalo, N. Y., October 16, 1894.

all sorts of contortions are seen until the parasites become dead and motionless. Meat may be digested artificially and the trichinæ liberated from their capsules by submerging it in water at 98°, to which hydrochloric acid and pepsin are added in the proportions found in the gastric juice. This is an efficient means of observing their motion. The capsules occupied by the trichinæ are usually oval and often much elongated. They may be perfectly round. The changes retrogressive—which they with their contents tend to undergo—are calcification, dark pigmentation and disintegration, varying from slight to complete loss of identity. I have observed trichinæ escaping from their capsules and have seen them advance and recede under the microscope. Dr. Hill has photographed them for me in this condition. In fresh specimens only empty capsules and liberated trichinæ are seen many times. I have sixty micro-photographs illustrating the pathology of trichinosis, which I will now have thrown upon the screen, and I will point out to you the conditions in particular which each photograph represents. These photo-micrographs have been made by Dr. Hill, of the University of Buffalo, from slides which I have referred to him.

In a study of the subject of trichinosis in the human I found 14 per cent. of a series of cases in the dissecting-rooms of the University of Buffalo invaded by this parasite. When a quantity of trichinæ not sufficient to produce death is ingested, they bore into the muscles even of remote parts, and I believe that many cases of chronic muscular rheumatism are due to the lodgment of this parasite in the muscles. In fact, the cases investigated are corroborative.

I have frequently observed trichinæ in adipose tissue and had them photographed in this position. Encapsulation here is very imperfect.

Recently an epidemic of trichinosis occurred in Halstadt, Belgium. There were thirty-nine cases, thirteen of which proved fatal. Old and obscure trichinæ cysts may similate Meischerian sacs, miliary tubercles, aggregations of fat globules and certain other conditions; from these they must be differentiated. Fifty thousand trichinæ may be present in a single ounce of pork. I have observed over 1,000 in a single slide.

I am limited to ten minutes for this synopsis, so will have to conclude. My paper will appear in full, with illustrations, in the Reports of the Bureau of Animal Industry of the United States Department of Agriculture.

